# The effects of a complex yoga-based intervention on healthy psychological functioning

**DOI:** 10.3389/fpsyg.2023.1120992

**Published:** 2023-03-30

**Authors:** Adam Koncz, Eszter Nagy, Barbara Csala, János Körmendi, Vera Gál, Csilla Suhaj, Csongor Selmeci, Ágota Selmeciné Bogdán, Szilvia Boros, Ferenc Köteles

**Affiliations:** ^1^Institute of Health Promotion and Sport Sciences, ELTE Eötvös Loránd University, Budapest, Hungary; ^2^Ádám György Psychophysiology Research Group, Budapest, Hungary; ^3^Institute of Psychology, ELTE Eötvös Loránd University, Budapest, Hungary; ^4^Doctoral School of Psychology, ELTE Eötvös Loránd University, Budapest, Hungary; ^5^Magyar Jóga Társaság/Hungarian Yoga Society, Veszprém, Hungary; ^6^Institute of Psychology, Károli Gáspár University of the Reformed Church in Hungary, Budapest, Hungary

**Keywords:** stress, interoceptive awareness, satisfaction with life, positive and negative affect, yoga-based intervention, spirituality

## Abstract

**Background:**

Yoga based interventions were found to have a positive impact on various indicators of psychological functioning, such as perceived stress, satisfaction with life, positive and negative affectivity. Additionally, such interventions improved bodily or interoceptive awareness and spirituality.

**Methods:**

The present study assessed the effects of a 3-month long complex yoga-based intervention compared to a passive control group in a Hungarian community sample. The final sample consisted of 44 intervention (Mage = 47.5, SD = = 8.76) and 29 control participants (Mage = 47.4, SD = 9.47). The aforementioned constructs were measured 1 week before and after the intervention.

**Results:**

The intervention reduced participants’ perceived stress (*p* = <0.001, *η_p_*^2^ = 0.153) and negative affectivity (*p* = 0.019, *η_p_*^2^ = 0.113), improved spirituality (*p* = 0.048, *η_p_*^2^ = 0.054) and various aspects of interoceptive awareness such as noticing (*p* = <0.001, *η_p_*^2^ = 0.169) attention regulation (*p* = <0.001, *η_p_*^2^ = 0.211), self-regulation (*p* = 0.002, *η_p_*^2^ = 0.190) body listening (*p* = 0.010, *η_p_*^2^ = 0.097), trusting (*p* = 0.026, *η_p_*^2^ = 0.070), but did not impact positive affectivity and satisfaction with life.

**Conclusion:**

A 3-months long complex yoga-based intervention has a positive impact on many aspects of healthy psychological functioning.

## 1. Introduction

Yoga is a complex mind–body approach (Iyengar, 1991), even though the majority of people in Western cultures considers it merely a physical activity (MacDonald, 2013; Sarbacker, 2014). Many experts claim that practicing yoga is a cost-effective way to improve mental health and quality of life (Büssing et al., 2012a; Shroff et al., 2017; Domingues, 2018; Csala et al., 2021).

Empirical findings largely support this view. For example, a number of intervention studies support the idea that yoga practice has a favorable impact on perceived stress after 8–16 weeks of practice (Smith et al., 2007; Gard et al., 2012; Bilderbeck et al., 2013; Schmalzl et al., 2018). For the most part, yoga interventions utilize a specific yoga tradition called hatha yoga. Classical hatha yoga is a practical discipline that includes, among others, the practice of physical postures (‘asanas’) and breath control (‘pranayama’) in order to facilitate meditation and attain spiritual goals (‘samadhi’; based on the Hatha Yoga Pradipika, an influential yogic text composed by Svatmarama in the 1400s; Mallinson, 2011). In Bilderbeck et al.’s (2013) study, in contrast with a passive control group, perceived stress and psychological distress decreased significantly after 10 weeks of practicing hatha yoga in a prison population. Gard et al. (2012) evaluated the outcome of a four-month-long residential program with Kripalu yoga (a branch of hatha yoga that emphasizes compassion and consciousness that allow the practitioner to observe events without judgement, a capacity much like mindfulness). It was found that participation predicted reduced stress and enhanced quality of life, and thus contributed to the well-being of young adults. In the study of Schmalzl et al. (2018), 8 weeks of yoga-based practices (YBP) focusing on movement and breath decreased perceived stress in young beginners. Based on the findings of Smith et al. (2007), hatha yoga is as effective in reducing stress as relaxation after ten weekly one-hour-long class. Moreover, when comparing advanced hatha yoga practitioners with beginners, the former reported considerably lower levels of stress (Brisbon and Lowery, 2011). Beyond perceived (self-reported) stress, a beneficial physiological effect, i.e., reduction of salivary cortisol, was also found (West et al., 2004; Schmalzl et al., 2018). According to the reviews of Ross and Thomas (2010) as well as Pascoe and Bauer (2015), yoga may cause these desirable effects by soothing the nervous system, i.e., by down-regulation of the hypothalamic–pituitary–adrenal axis and the sympathetic nervous system. Yoga might be beneficial in this regard for vulnerable populations as well, such as elderly people or people suffering from mental disorders characterized by excessive amounts of stress (Bonura and Pargman, 2009; Doria et al., 2015; Jindani et al., 2015).

Concerning positive affectivity like joyfulness or alertness, and negative affectivity that refers to unpleasurable feelings (Watson, 1988), yoga might also have a beneficial effect (Meissner et al., 2016). Although a recent review of Domingues (2018) found contradictory results with respect to affectivity, some evidence supports the idea that increasing the amount of time spent with yoga practice leads to increase of positive affectivity and decrease of negative affectivity (Meissner et al., 2016).

Although an enhanced satisfaction with life due to yoga practice could also be expected, empirical results have not been conclusive so far. Participants reported marginally increased satisfaction with their life in three yoga-based intervention studies (Impett et al., 2006; Elavsky and McAuley, 2007; Mathad et al., 2017). In another study, although quality of life, as assessed with the Satisfaction with Life Scale (SWLS; Diener et al., 1985), improved for epilepsy patients practicing yoga, this effect was not shown with the short version of the World Health Organization Quality of Life (WHOQOL-BREF; Harper et al., 1998; Lundgren et al., 2008).

Spirituality (religious or non-religious) refers to a pursuit of meaning in life and connection to transcendental phenomena, and involves specific values and beliefs related to this transcendental view of life (Sessanna et al., 2007). MacDonald (2013) highlighted the importance of including spirituality in yoga research, as its benefits for mental health appear evident. Yoga practice increased self-rated spirituality in a few studies (Büssing et al., 2012b; Gaiswinkler and Unterrainer, 2016). Likewise, 5 days were enough to elicit increased spiritual growth in the study of Braun et al. (2012) in a residential, Kripalu yoga-based program. Based on the systematic review of Csala et al. (2021), both quantitative and qualitative studies suggest that yoga, especially regular practice, positively affects several aspects of spirituality. It is also worth mentioning that yoga practitioners exhibited the highest level of spirituality when compared to practitioners of martial arts of Eastern origin (aikido, judo) and controls (Western sports; Szabolcs et al., 2021). However, there are also a few studies with conflicting results about whether yoga increases spirituality, and if so, how. Two similar, hatha yoga-based interventions designed for cancer patients, yoga did not increase spirituality. In Cramer et al.’s (2016) 10-week-long yoga program for patients with colorectal cancer, no significant group differences emerged with respect to spiritual well-being; on the other hand, although practicing yoga for 12 weeks did not increase spirituality in breast cancer patients, its initial level was more preserved compared to waiting list controls, based on Moadel et al.’s (2007) work. In both cases, however, adherence was compromised; moreover, drop-out rate was also high in Cramer et al.’s (2016) program. In Csala et al.’s (2020) study, university students, who participated in a 10-week-long beginner hatha yoga program, generally reported higher levels of spiritual connection, regardless of whether they received verbal cues referring to spirituality or physical performance from their instructor. According to the authors’ explanation, yoga might exert its effect primarily through bodily processes and not through verbal instructions.

It is often claimed that yoga can also affect body awareness, i.e., the perceived ability to detect bodily changes and to focus on them. Enhancement of various aspects of body awareness or interoceptive awareness is considered a common mechanism of action for mind–body therapies (Mehling, 2016). In a cross-sectional study Daubenmier (2005) found higher body awareness in healthy adult women practicing yoga compared to aerobic exercisers and to those who did neither. Rani and Rao (1994) found that a 3-month hatha yoga program (daily 1 h practicing) increased body awareness among healthy adults. Delaney and Anthis (2010) also found in a cross-sectional study that mind–body emphasis could be associated with higher body awareness. In two cross-sectional studies, Tihanyi and colleagues examined advanced yoga practitioners. These participants showed significantly higher levels of body awareness compared to people who do other sports (kung-fu, aerobics, ballroom dance; 2016a); additionally, weekly frequency of yoga practice among advanced practitioners correlated positively and significantly with body awareness, and the latter also proved to be a mediator between yoga practice and well-being (2016b). Similarly, in Impett et al.’s (2006) study, the more participants practiced yoga, the more they were likely to report an increase in their body awareness (although the effect was only marginal in this case). However, no changes in body awareness were reported by Csala et al. (2020) after a 10 week long (1.5 h per week) yoga course.

Overall, practicing yoga has been associated with improvement in several psychological factors that potentially positively impact an individual’s quality of life to a considerable extent. However, current literature is not without conflicting results, especially regarding how yoga influences affectivity, the level of general satisfaction with life and spirituality. In some cases, the inconclusive nature of the question at hand may derive from the use of different measurements instruments (as in case of the Satisfaction with Life Scale vs. WHOQOL), population properties (as in case of the cancer patients who may face extreme existential crises that hinders them from experiencing spiritual well-being), or the lack of sufficient evidence [as mentioned in the review of Domingues (2018) examining yoga and positive mental health indicators]. Despite the negative results, the overwhelming majority of studies do support the notion that yoga is beneficial to body and mind, so positive outcomes can reasonably be expected. Nevertheless, current literature would still benefit from more information on these subjects from healthy populations.

A new feature of this study, compared to others available in the literature, is that it incorporates other psychoeducational elements rather than just yoga, making it more easily acceptable by a community sample. An important contribution is that very few studies have examined the effects of yoga interventions on body awareness or spirituality and rather used shorter interventions. Finally, in the present study the yoga intervention is well defined compared to the previous ones available in the literature (Csala et al., 2021).

The aim of the present study was to investigate the impact of a complex 3-month yoga-based intervention on participants’ perceived stress, affectivity, satisfaction with life, spirituality and body awareness. Based on the empirical findings, the following hypotheses were formulated and tested: Yoga practice can decrease negative mental states such as (i) perceived stress and (ii) negative affectivity. Additionally, yoga leads to (iii) increased positive affectivity and (iv) higher satisfaction with life. Also, we expected an increase in (v) spirituality and (vi) body awareness.

## 2. Materials and methods

### 2.1. Study approval

The present study was approved by the Research Ethics Committee of the Faculty of Education and Psychology at ELTE Eötvös Loránd University under the registration number 2021/44 on 21 April 2021.

### 2.2. Participants

**Figure 1:**
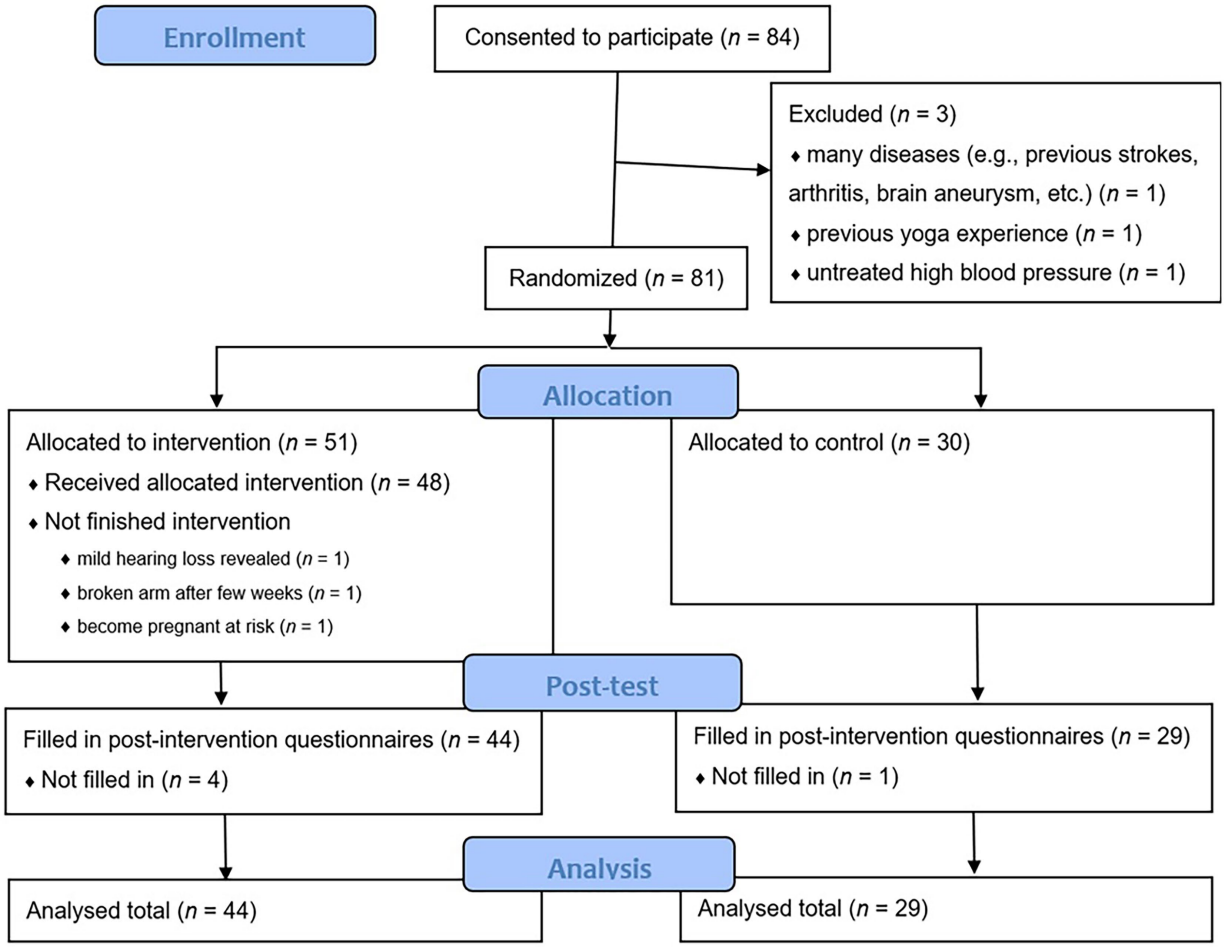
Number of participants at each point of time.

Participants were recruited through public lectures in Veszprém, Hungary and social media (Facebook). Recruitment was carried out by the AHIMSZA Yoga Center. Inclusion criteria were (1) lack of cardiovascular, locomotor, or other disease that would have made regular yoga practice risky (participants were examined by a sports medicine physician with extensive experience in yoga), and (2) no previous experience with yoga. Eighty four individuals agreed to participate; the final sample consisted of 73 participants. The intervention group consisted of 44 participants, whose mean age was 47.5 (SD = 8.76) years, ranging between 30 and 63; 44 were female (100%), and 84% of them had a higher level of education while 16% have a secondary level. The control group consisted of 29 participants, mean age was 47.4 (SD = 9.47) years, ranging between 30 and 64; 24 were female (83%), and 83% have a higher while 17% have a secondary level of education. (Figure 1 shows the total number of participants at various steps of recruitment and intervention). There were no significant differences between the control and experimental group in terms of age [*t*(71) = 0.113, *p* = 0.910, *d* = 0.239], sex [*χ*^2^(1, *N* = 73) = 0.178, *p* = 0.674], and educational qualification (*W* = 646.500, *p* = 0.888). Weekly frequency of home practice for the intervention group was on average 4.38 (SD = 1.47).

### 2.3. Design

In this study a quasi-randomized design was applied in order to maintain the desired ratio of high schoolteachers and local citizens (1:3; see below). Eligible participants were assigned to the passive control (receiving a 5,000 HUF ~ 13 EUR voucher as an incentive for their participation) or to the experimental group. For both categories, i.e., teachers and citizens, nicknames of the participants were written on slips of paper, and these tags were drawn from a bag by one of the authors (FK). The complex yoga-based intervention was delivered and thus data were collected in three waves for three independent groups of participants; in the first wave, there was no control group due to the requirements of the grant the project based on (see below). The participants of the intervention group were asked to send a Facebook message after each home practicing session. Pre- and post-test questionnaires were filled in 1 week before and after the interventions. Beyond the questionnaires, physiological and physical measurements were also carried out before and after the intervention.

### 2.4. Intervention materials

This study reports the psychological aspects of a complex yoga program designed and conducted by the Hungarian Yoga Teachers Association (HYTA) and the AHIMSZA Yoga Center, Veszprém, Hungary for primary and secondary school teachers and local citizens of Veszprém. The original project was supported by the EU (grant number: TOP-7.1.1-16-H-073-5.2). Aims of the project were (1) the dissemination of knowledge on healthy lifestyle among the citizens of Veszprém, a Hungarian city, (2) promotion of lifestyle changes, (3) creation of supportive small communities, especially with the inclusion of primary and secondary school teachers. Secondary purpose of the program was that schoolteachers involved can set up small groups and share their knowledge about healthy lifestyle within schools. The program was a 12 week-long training focusing on three major topics: 5 scientific lectures on health and healthy lifestyle, 12 practical yoga classes, and 10 club meetings. Face-to-face yoga sessions took place 1 session/per week and built on each other. Beyond these sessions, starting and keeping a regular home practice was a main element of the program (i.e., participants were explicitly encouraged to practice alone at home, see below). The aforementioned practical yoga classes were held for 1.5 h and contained physical movements with body awareness and breath synchronization, breathing exercises, and relaxation. Class plans were based on Iyengar yoga, Satyananda yoga, and yoga of the Kaivalyadhama Institute, India. Yoga classes included various postures and exercises, such as neck and shoulder movements, cat and cow pose, downward facing dog, standing poses, spine twists, leg raising exercises in supine position, prone positions, sitting poses, and hand half inversions. Also lying belly breathing exercise and relaxation in corpse position (Savasana) were parts of the classes (for more details, see Appendix A). For practical yoga classes, the intervention group was divided into two subgroups, i.e., schoolteachers and civil workers. Teacher participants were less in number to provide them more space and professional attention in order to prepare them to bring their knowledge into schools. The yoga classes were delivered by certified yoga instructors of HYTA. Besides these classes, students received written exercise series and video material for home practice and were asked to practice every day and to record the number of occasions they practiced per week. There were six series of yoga exercises that build on each other (increasing level, based upon the practical yoga classes). In addition to these, students received pranayama exercise series, too. These basic practice series were compiled based upon six yoga traditions, namely Gitananda, Krishnamacharya, Iyengar, Satyanada, Kaivalyadhama Institute, and The Yoga Institute, and mainly aimed at reducing stress. Gitananda approached yoga as a ‘way of life’, rather than an activity we sustain for short periods of time; he also placed an emphasis on the moral teachings of yoga, and a step-by-step, slow mastering that does not strain the body (Bhavanani, 2012). Krishnamacharya reformed hatha yoga, especially in relation to its sequencing: he separated asanas by special, dynamic sequences with controlled breathing and eye focus (Ruiz, 2001). Iyengar popularized a’modern’, postural approach to yoga, which aligned more with the needs of Western, secular society, primarily focusing on asana practice with clear instructions in order to improve body image, reduce stress and understand yoga from a cause and effect perspective (e.g., use yoga as a mean to lose weight or ease back pain; Lea, 2009). Satyanada’s tradition incorporates hatha yoga, the use of sound, relaxation, and emphasizes daily spiritual practice; it is also more social and accepting of experiences related to modern, worldly life than classical yoga (Persson, 2010). Kaivalyadhama Institute and The Yoga Institute are two research centers in India that have conducted research and provided scientific ground for yoga since 1924 and 1918, respectively (The Oldest Scientific Yoga Research Institute in the World: Kaivalyadhama, n.d.; World’s Oldest Yoga Institute: The Yoga Institute, n.d.). Beyond yoga sessions, scientific lectures and club meetings were led by another instructor of HYTA; here, no subgroups were applied. Scientific lectures on health started after the second practical yoga session and were repeated on a weekly basis (ending at around half time of the program). Order of the sessions is presented in Appendix A. These lectures were held online, topics were as follows: healthy sleeping habits for hormonal balance (1), physiological and psychological effects of nutrition, developing a healthy diet (2), the importance of stress-management and relaxation (3), the relevance of breathing in stress-management, physiology of breathing (4), the role of asana practice on developing healthy body awareness, effects of asanas on physiology (i.e., cardiovascular, neural, and hormonal functioning) (5). Club meetings were held around every second week right before or after the practical yoga session (Appendix A). Club meetings aimed to establish a regular home practice and offered time and place for participants to share their experiences with each other and the yoga teachers and to discuss questions regarding their home practice or any other ones. Club meetings were designed to promote gaining new information and enhance motivation to self-practice and keeping a healthy lifestyle. Three of the ten club meetings took place after the post-intervention measurement to help the participants maintain their home practice and healthy lifestyle habits even after the program.

### 2.5. Questionnaires

#### 2.5.1. Positive and negative affect schedule

The Hungarian translation (Gyollai et al., 2011) of the short form (I-PANAS-SF; Thompson, 2007) of the original Positive and negative affect schedule was used (Watson, 1988). This version contains two scales consisting of 5–5 items, that are rated on a five-point scale ranging from “very slightly or not at all” to “extremely.” The Positive Affect scale assesses how active, vigilant, or enthusiastic the person feels, whereas the Negative Affect scale measures negative moods of the respondent like guilt, nervous or upset. Higher scores represent higher levels of negative and positive affect. The Cronbach’s alpha values in the present study in the pre-test were 0.653 for negative and 0.709 for positive affectivity, respectively; while in the post-test it was 0.724 for negative and 0.749 for positive affectivity, respectively.

#### 2.5.2. Multidimensional assessment of interoceptive awareness

Various aspects of interceptive body awareness were measured with the Hungarian version (Ferentzi et al., 2020) of MAIA, a 32-item long questionnaire. It consists of eight scales: Noticing, Not-Distracting, Not-Worrying, Attention Regulation, Emotional Awareness, Self-Regulation, Body Listening, and Trusting (Mehling et al., 2012). Items are rated on a 6-point Likert scale ranging from “never” to “always.” Higher values indicate higher levels of the respective construct. Cronbach’s alphas in the pre-test were 0.814 for Noticing, 0.559 for Not-Distracting, 0.748 for Not-Worrying, 0.916 for Attention Regulation, 0.910 for Emotional Awareness, 0.878 for Self-Regulation, 0.893 for Body Listening, and 0.920 for Trusting. In the post-test, Cronbach’s alphas were 0.821 for Noticing, 0.564 for Not-Distracting, 0.731 for Not-Worrying, 0.908 for Attention Regulation, 0.868 for Emotional Awareness, 0.879 for Self-Regulation, 0.902 for Body Listening, and 0.893 for Trusting.

#### 2.5.3. Perceived stress scale (PSS)

This questionnaire aimed to measure how often the respondent felt their life overloaded, or felt he/she had difficulty coping with certain life situations in the last month (Cohen et al., 1983). The short version of the questionnaire consists of 4 items, that are rated on a 5-point Likert scale ranging from “never” to “very often.” Higher scores refer to higher levels of perceived stress. In the current study, the Hungarian version of the scale was used (Stauder and Konkoly Thege, 2006); Chronbach’s alpha was 0.716 in the pre- and 0.764 in the post-test.

#### 2.5.4. Satisfaction with life scale (SWLS)

This questionnaire measures global life satisfaction. It consists of five items rated on a 7-point Likert scale (from “strongly disagree” to “totally agree”); higher scores refer to higher levels of satisfaction (Diener et al., 1985; Martos et al., 2014). Cronbach’s alpha was 0.864 on pre-test and 0.818 on post-test.

#### 2.5.5. Spiritual connection questionnaire (SCQ-14)

The questionnaire measures the subjective feeling of connection with people and the universe, additionally the pleasure caused by the sense of connection (Wheeler and Hyland, 2008; Csala and Köteles, 2021). The questionnaire consists of 14 items rated on a seven-point Likert scale ranging from “definitely not agree” to “definitely agree.” Higher scores indicate higher level of spirituality. In the present study, Cronbach’s alphas were 0.753. on pre-test and 0.951 in case of post-test measurement.

### 2.6. Statistical analyses

Statistics was implemented using JASP v0.16.4 software (JASP Team, 2022). Data outside the range of the 1.5*interquartile range from the 25 or 75 percentile were considered outliers and removed (Vinutha et al., 2018). To compare the intervention and control group’s baseline values in the case where the data of the two groups to be compared were normally distributed, independent samples *t*-tests were performed and Cohen’s *d*’s were reported. Based on Cohen’s benchmarks, *d* = 0.2 refers to small *d* = 0.5 refers to medium, and *d* = 0.8 refers to large effect size (Cohen, 1988). Mann–Whitney tests were carried out where data did not meet normal distribution. Hypotheses were tested with 2 × 2 mixed analyses of variance (ANOVA) where time (baseline, post-intervention) was used as within-subjects factor and condition (yoga, control) as between-subjects factor regardless of normality, because ANOVA’s robustness allows the violation of such assumptions (Blanca Mena et al., 2017). Effect sizes of ANOVA are reported as partial eta squared (*η_p_*^2^) which is considered as small 0.0099 medium 0.0588, and large 0.1379 based on Cohen’s benchmarks (Cohen, 1988).

## 3. Results

### 3.1. Baseline differences in outcome variables

No significant difference was found between the control and experimental group for any of the outcome variables but positive affectivity [*t*(69) = 2.018, *p* = 0.048, *d* = 0.487]. Analysis indicated homogeneity of the two groups for negative affectivity (*U* = 744.50, *p* = 0.092, *d* = 0.237), MAIA Noticing (*U* = 607.00, *p* = 0.986, *d* = −0.003), MAIA Not distracting [*t*(71) = 0.566, *p* = 0.573, *d* = 0.135], MAIA Not Worrying [*t*(71) = 0882, *p* = 0.381, *d* = 0.211], MAIA Attention Regulation (*U* = 726.50, *p* = 0.320, *d* = 0.139), MAIA Emotional Awareness (*U* = 642.00, *p* = 0.836, *d* = 0.030), MAIA Self-Regulation (*U* = 268.50, *p* = 0.580, *d* = −0.096), MAIA Body Listening (*U* = 705.00, *p* = 0.452, *d* = 0.105), MAIA Trusting (*U* = 697.50, *p* = 0.504, *d* = 0.093), PSS (*t*(71) = −0.040, *p* = 0.968, *d* = −0.010), SWLS (*t*(70) = −0.312, *p* = 0.756, *d* = 0.075), and SCQ14 (*U* = 599.00, *p* = 0.664, *d* = −0.061).

### 3.2. Effects of the intervention

#### 3.2.1. Positive and negative affect schedule

**Figure 2:**
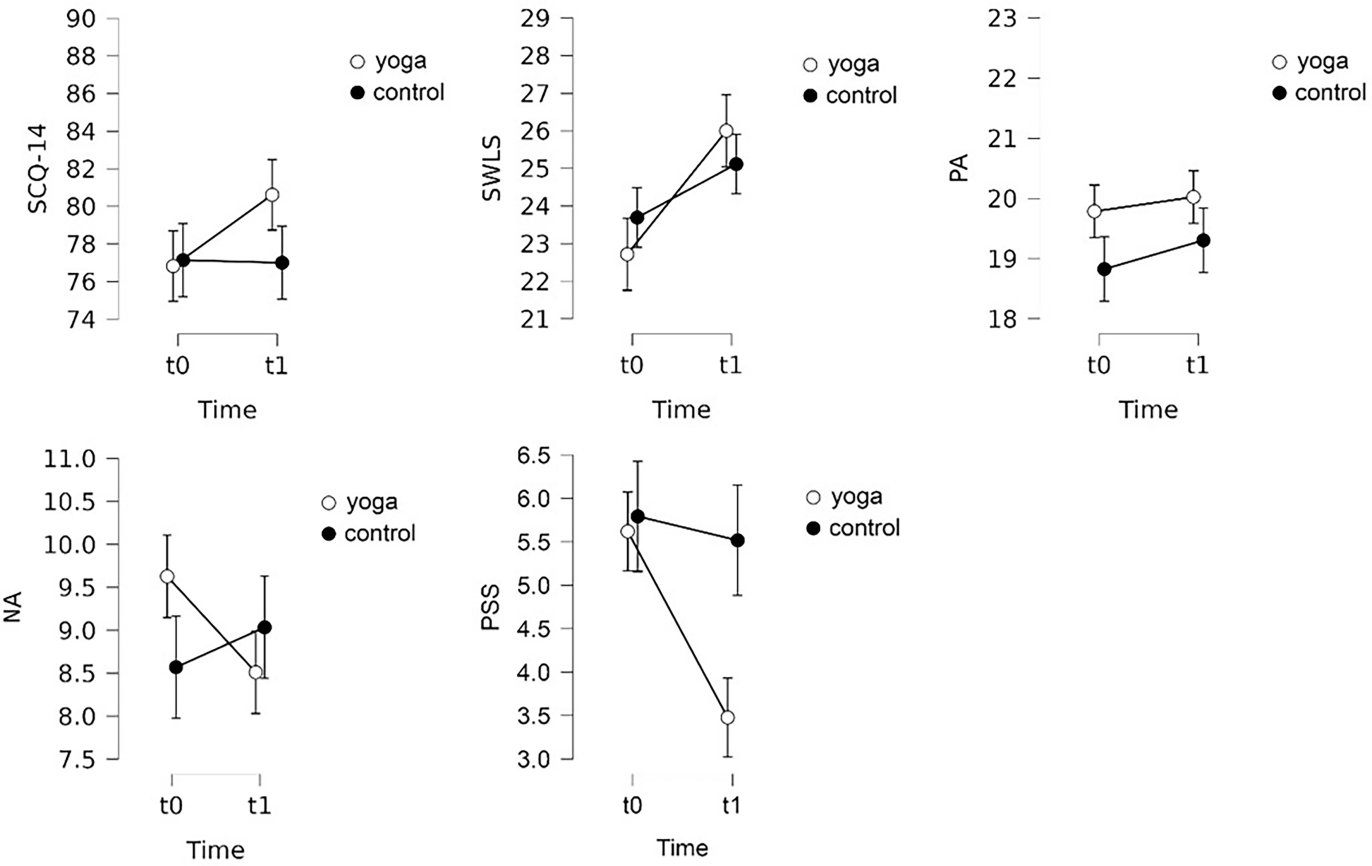
Descriptive plots of the Spiritual Connection Questionnaire (SCQ-14), Satisfaction with Life Scale (SWLS,) Positive and Negative Affect Schedule’s Positive Affect (PA) and Negative Affect (NA) scales, and the Perceived Stress Scale (PSS). Error bars indicate 95% confidence intervals.

For positive affectivity no significant main effect of time or condition, and no significant time x condition interaction were found. In contrast, for negative affectivity, although no significant main effect of time or condition was found again, but a significant time x condition interaction, that is a decrease of negative affectivity in the experimental group compared to the control participants, was revealed (for descriptive statistics see Table 1 and Figure 2, for test statistics see Table 2).

**Table 1:** Descriptive statistics of the assessed variables before and after the intervention.

Scale	Pre-test				Post-test			
	Intervention		Control		Intervention		Control	
	Mean (SD)	*n*	Mean (SD)	*n*	Mean (SD)	*n*	Mean (SD)	*n*
**PANAS**								
Positive affect	19.79 (2.33)	42	18.83 (1.95)	23	20.02 (2.51)	42	19.30 (1.89)	23
Negative affect	9.63 (2.69)	43	8.57 (2.65)	28	8.51 (2.94)	43	9.04 (2.87)	28
**MAIA**								
Noticing	3.41 (1.01)	42	3.44 (0.81)	28	4.03 (0.85)	42	3.53 (0.68)	28
Not-distracting	2.62 (0.96)	43	2.51 (0.85)	29	2.55 (0.86)	43	2.68 (0.92)	29
Not-worrying	2.30 (1.18)	44	2.07 (0.89)	29	2.52 (1.12)	44	1.97 (0.98)	29
Attention regulation	2.45 (1.07)	44	2.28 (0.89)	29	3.26 (0.79)	44	2.34 (0.94)	29
Emotional awareness	3.70 (0.93)	39	3.56 (0.92)	28	4.24 (0.60)	39	3.71 (0.73)	28
Self-regulation	2.45 (1.15)	32	2.63 (1.01)	18	3.60 (0.71)	32	3.01 (0.82)	18
Body listening	2.50 (1.15)	41	2.22 (1.16)	27	3.47 (0.77)	41	2.58 (0.90)	27
Trusting	3.00 (1.14)	43	2.87 (1.02)	28	3.95 (0.80)	43	3.32 (093)	28
**PSS**								
Total score	5.62 (2.20)	42	5.79 (1.93)	29	3.48 (1.95)	42	5.52 (2.53)	29
**SWLS**								
Total score	22.71 (5.56)	42	23.69 (4.62)	26	26.00 (3.48)	42	25.12 (3.40)	26
**SCQ**								
Total score	76.82 (14.84)	44	77.14 (16.94)	29	80.61 (13.73)	44	77.00 (18.55)	29

PANAS = Positive and Negative Affect Schedule, MAIA = Multidimensional Assessment of Interoceptive Awareness, PSS = Perceived Stress Scale, SWLS = Satisfaction with Life Scale, SCQ = Spiritual Connection Questionnaire.

**Table 2:** Effects of the intervention on positive and negative affectivity, interoceptive awareness, perceived stress scale, satisfaction with life and spiritual connection.

Scale	Time				Condition				Time × condition			
	*F*	*df*	*p*	*η_p_* ^2^	*F*	*df*	*p*	*η_p_* ^2^	*F*	*df*	*p*	*η_p_* ^2^
**PANAS**												
Positive affect	2.095	1,63	0.153	0.032	2.492	1,63	0.119	0.038	0.235	1,63	0.629	0.004
Negative affect	1.501	1,69	0.225	0.021	0.182	1,69	0.671	0.003	8.821	1,69	0.019*	0.113
**MAIA**												
Noticing	12.332	1,68	<0.001*	0.154	0.893	1,68	0.348	0.013	13.826	1,68	<0.001*	0.169
Not-distracting	0.171	1,70	0.680	0.002	0.001	1,70	0.970	0.000	0.955	1,70	0.332	0.013
Not-worrying	0.247	1,71	0.621	0.003	2.913	1,71	0.092	0.039	1.905	1,71	0.172	0.026
Attention regulation	25.475	1,71	<0.001*	0.264	6.976	1,71	0.010*	0.089	18.982	1,71	<0.001*	0.211
Emotional awareness	12.213	1,65	<0.001*	0.158	3.804	1,65	0.055	0.055	3.977	1,65	0.050	0.058
Self-regulation	45.726	1,48	<0.001*	0.488	0.650	1,48	0.424	0.013	11.278	1,48	0.002*	0.190
Body listening	33.480	1,66	<0.001*	0.337	7.142	1,66	0.009*	0.098	7.078	1,66	0.010*	0.097
Trusting	40.684	1,69	<0.001*	0.371	3.258	1,69	0.075	0.045	5.169	1,69	0.026*	0.070
**PSS**												
Total score	20.925	1,69	<0.001*	0.233	6.141	1,69	0.016*	0.082	12.468	1,69	<0.001*	0.153
**SWLS**												
Total score	24.305	1,66	<0.001*	0.269	0.002	1,66	0.963	0.000	3.803	1,66	0.055	0.054
**SCQ**												
Total score	3.505	1,71	0.065	0.047	0.205	1,71	0.652	0.003	4.054	1,71	0.048*	0.054

PANAS = Positive and Negative Affect Schedule, MAIA = Multidimensional Assessment of Interoceptive Awareness, PSS = Perceived Stress Scale, SWLS = Satisfaction with Life Scale, SCQ = Spiritual Connection Questionnaire; ^*^ = *p* < 0.05, *F* = *F* value of the repeated measures ANOVA, *η_p_*^2^ = partial ETA squared.

#### 3.2.2. Multidimensional assessment of interoceptive awareness

**Figure 3:**
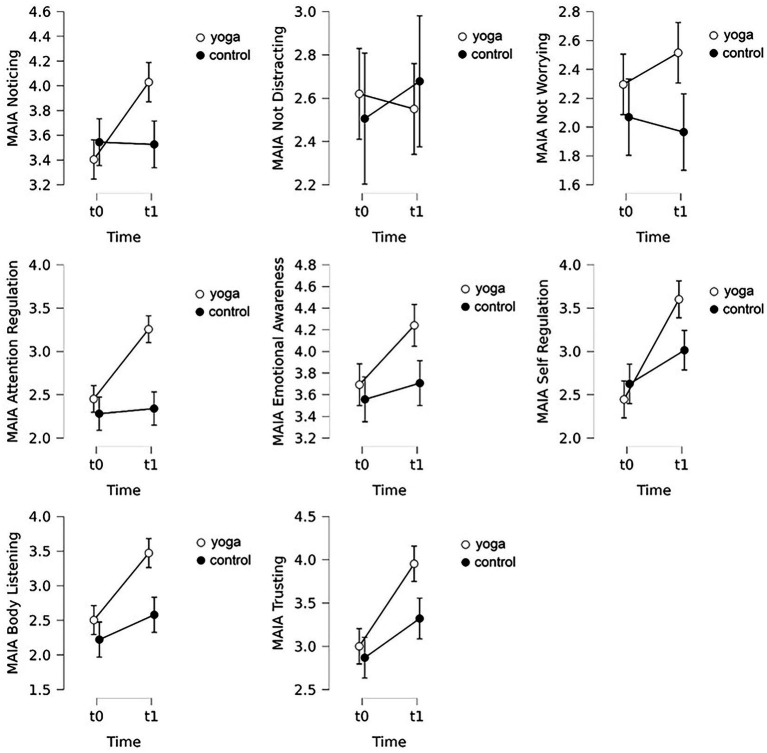
Descriptive plots of the scales of the Multidimensional Assessment of Interoceptive Awareness (MAIA) questionnaire. Error bars indicate 95% confidence intervals.

In case of Noticing, a significant main effect of time (an increase in the scores) but no main effect of condition was found. The time x condition interaction was significant, the experimental group improved to a larger extent than the control group. Concerning the Not-Distracting and the Not-Worrying scales, no significant main effect of time or condition and no time x condition interaction were found. Regarding Attention Regulation, a significant main effect of time was found (scores increased), besides main effect of condition was also significant. Finally, the time x condition interaction was also significant, that is the increase in the experimental group was larger than the control group’s change. In case of Emotional Awareness, again a significant main effect of time was detected that is an increase in the scores, but no significant main effect of condition and significant time x condition interaction were found. Regarding Self-Regulation, again, significant main effect of time thus an increase on the scores on this scale, but no main effect of condition was found. The interaction between time x condition was also significant; the experimental group improved more that the control group. Body Listening increased from pre- to post-intervention, so the main effect of time was significant, additionally a main effect of condition was also found, and the time x condition interaction was also significant (again, the experimental group improved to a greater extent). Finally in case of Trusting, significant main effect of time was found, again an increase was detected, whereas the main effect of condition was not significant. Interaction between time and condition reached the level of significance, a larger increase in the experimental group was found again (for descriptive statistics see Table 1 and Figure 3, for test statistics see Table 2).

#### 3.2.3. Perceived stress scale

Regarding Perceived Stress main effect of both time and condition were significant, that is an overall decrease in perceived stress. Additionally, a significant time x condition interaction was found, experimental participants’ perceived stress decreased to a larger extent that that of control participants (for descriptive statistics see Table 1 and Figure 2, for test statistics see Table 2).

#### 3.2.4. Satisfaction with life scale

In case of this scale time had a significant main effect, i.e., satisfaction with life increased in the entire sample, but neither a significant main effect of condition nor significant time x condition interaction was found (for descriptive statistics see Table 1 and Figure 2, for test statistics see Table 2).

#### 3.2.5. Spiritual connection questionnaire

In case of spiritual connection, no main effect of time and condition was found but the time x condition interaction was significant. Compared to the control group, spiritual connection increased in the experimental group (for descriptive statistics see Table 1 and Figure 2, for test statistics see Table 2).

## 4. Discussion

In a controlled intervention study, a complex hatha yoga intervention with a duration of 3 months led to an increase in spirituality, in the perceived tendency to notice body changes (MAIA noticing), the ability to direct attention to them (MAIA attention regulation), using them as means of self-regulation (MAIA self-regulation), and considering them a reliable and important indicator of the internal state (MAIA trusting and body listening), and a decrease in negative affect and perceived stress. However, no impact on positive affect, satisfaction with life, and some of the variables measured by the MAIA questionnaire such as Emotional Awareness, and the ability to distract attention from unpleasant body sensations (Not distracting) and their affective concomitants (Not worrying) were found.

Both perceived stress and negative affect decreased during the yoga intervention. The former outcome is in line with findings reported in the literature (West et al., 2004; Smith et al., 2007; Bonura and Pargman, 2009; Brisbon and Lowery, 2011; Gard et al., 2012; Bilderbeck et al., 2013; Doria et al., 2015; Jindani et al., 2015; Schmalzl et al., 2018), and further supports the notion that yoga can be an effective intervention for the management of stress. Concerning the finding about reduced negative affectivity, our results may contribute to the dissolution of contradictions implied by the review of Domingues (2018) and viewing yoga as a tool for soothing negative affective states.

Our finding about enhanced spirituality due to yoga practice also fits nicely into existing literature (Braun et al., 2012; Büssing et al., 2012b; Gaiswinkler and Unterrainer, 2016; Csala et al., 2021; Szabolcs et al., 2021). As the majority of studies with healthy populations, and now ours too, support the notion that enhanced spirituality and yoga are associated, it is likely that the two intervention studies that did not yield similar results (Moadel et al., 2007; Cramer et al., 2016) reached different conclusions because of the properties of a special population.

However, contrary to our preliminary assumptions, we could not report consistent results about the relationship between yoga practice and various aspects of interoceptive awareness. Yoga positively affected the ability to notice, attend to, listen to, and place trust in relevant body sensations, and even regulate one’s state (e.g., reduce tension, reach a state of calmness) based on them. Meanwhile, yoga did not seem to lead to more insight into emotional experience related to body sensations, nor did it lead to less worrying about and less distracting oneself from unpleasant body sensations. The lack of relationship between these aspects of body awareness and yoga is intriguing. Similarly, to our results, Bornemann et al. (2015) did not find significant changes in the aforementioned aspects; they reason that training bodily focus leads to better attention and clarity about bodily sensations and affective states, but enhanced bodily focus in itself is not sufficient for further processing the unpleasant sensations, cognitions or emotions that may arise. In Floyd et al.’s (2022) study, ten sessions of yoga practice did not elicit improvements in the Not-Distracting and Not-Worrying subscales of MAIA either. It is possible that the reported associations (Daubenmier, 2005; Delaney and Anthis, 2010; Tihanyi et al., 2016a,b) were valid only cross-sectionally, e.g., as a consequence of self-selection. The questionnaire we used captured more dimensions of the broad construct of interoceptive awareness, thus there was more room for differential results than in case of studies that used questionnaires (typically the BAQ) with a single composite score (Rani and Rao, 1994; Daubenmier, 2005; Impett et al., 2006; Tihanyi et al., 2016b). It may not even be reasonable to draw a parallel between the aforementioned instruments, as Vig et al. (2022) recently found that correlations between the BAQ and the MAIA scales Not Worrying and Not Distracting are virtually non-existent, as well as only weak to moderate associations have been found with the remaining six subscales. Consequently, the authors suggest that these instruments cannot be used interchangeably, as they presumably do not measure the same underlying construct. It is also worth noting that the subscales Not Worrying and Not Distracting were only weakly related to a general factor of interoception in a study that aimed to examine the originally proposed factor structure of the MAIA (Ferentzi et al., 2020). Based on these findings, we could conclude that regular yoga practice does not improve the ability to regulate body-related distress. However, an improvement was found for five scales out of six that belong to the general interoception factor.

Interestingly, positive affectivity and satisfaction with life were not impacted by the yoga intervention. Contrary to the findings supporting the existence of a positive association between yoga and positive affectivity (Impett et al., 2006; Kiecolt-Glaser et al., 2010; Tihanyi et al., 2016a), West et al. (2004) also found no increase in positive affectivity after yoga practice. In their study, positive affect increased in people practicing African dance, but did not change in the yoga group, while negative affect decreased in both conditions. Also, salivary cortisol increased in the dance group, and decreased in the yoga group. West et al. (2004) drew the conclusion that although both activities are beneficial for the psyche, the underlying physiological processes differ considerably. It is also possible, that other aspects, such as forms or frequency of practice are of key importance. Meissner et al. (2016) found that when practitioners who originally took a Western approach to yoga practiced more traditionally (every day in the morning) for 2 weeks, their positive affectivity did actually increase. Tihanyi et al.’s (2016a) findings point to this direction too, as in their study weekly frequency of yoga practice was associated with higher levels of positive affect. These results and ours pose more questions about how yoga and positive affectivity really relate to each other.

As far as life satisfaction is concerned, one possible explanation is that domain-general life satisfaction is more stable over this time interval—there is a high year-to-year correlation, and major life changes and relatively stable intra-and interpersonal resources are more likely to account for the variance, exerting their dominating effect throughout years (Ehrhardt et al., 2000).

In summary, the yoga intervention reported in this study had a positive impact on many aspects of healthy psychological functioning, such as perceived stress, negative affectivity, spirituality, and various indicators of self-reported interoception. These findings, that support the idea that regular yoga practice can be beneficial for mental health (Büssing et al., 2012a; Shroff et al., 2017; Domingues, 2018), are certainly worthy of further scientific investigation.

These results can contribute to improving the well-being of the participants, and the participating teachers can also contribute to the healthy lifestyles of their students by passing on the knowledge acquired. Changes in the lifestyle and psychological well-being of these students of the participating teachers may also be worth investigating in the future.

## 5. Limitations

It is important to note that participation in the intervention took place in three waves and there was no corresponding control group in the first wave. The practical yoga classes were held separately for schoolteachers and civilian workers. Subjects were mainly women and high level of motivation and interest of participants clearly limits the generalizability of the findings. The intervention as a whole was studied and compared to an inactive group; therefore, the contribution of certain components (e.g., physical activity, education, social factors) cannot be assessed. Weekly frequency of home practice was self-reported. The use of different yoga traditions poses a methodological problem in our study, which often occurs in yoga research, because the distinction between these traditions is not clearly defined nor well documented (Elwy et al., 2014; Park et al., 2014, 2018; Sullivan et al., 2018; Csala et al., 2021). However, all of these traditions can be traced back to, and are fundamentally related to hatha yoga. Additionally, because of the passive control condition participants were not blind regarding their grouping thus the expectations of the experimental participants could have an impact on the results in particular because we used self-report measures. In case of measured constructs, spirituality is not a well-defined concept and there is no one universally accepted definition or measurement method for it. Finally, the low internal consistency of the MAIA not distracting scale (both at pre- and post-intervention) renders the respective findings difficult to interpret.

## 6. Conclusion

In summary, a complex 3-month-long yoga-based intervention was able to decrease negative affect and perceived stress, increase spirituality and many aspects of interoceptive awareness.

## Data availability statement

The raw data supporting the conclusions of this article will be made available by the authors, without undue reservation.

## Ethics statement

The studies involving human participants were reviewed and approved by Research Ethics Committee of the Faculty of Education and Psychology at ELTE Eötvös Loránd University. The patients/participants provided their written informed consent to participate in this study.

## Author contributions

FK contributed substantially to the conception, design, interpretation of data, and provided feedback in order to improve the manuscript. AK, EN, FK, and BC prepared the manuscript. FK and AK conducted the statistical analyses. CSe and ÁB set up and implemented the intervention. JK, VG, CSu, and SB substantially contributed to the data collection. All authors contributed to the article and approved the submitted version.

## Funding

This research was supported by the Hungarian National Scientific Research Fund [K124132, K137724].

## Conflict of interest

The authors declare that the research was conducted in the absence of any commercial or financial relationships that could be construed as a potential conflict of interest.

## Publisher’s note

All claims expressed in this article are solely those of the authors and do not necessarily represent those of their affiliated organizations, or those of the publisher, the editors and the reviewers. Any product that may be evaluated in this article, or claim that may be made by its manufacturer, is not guaranteed or endorsed by the publisher.
