# Supplementary material for: The effects of a complex yoga-based intervention on healthy psychological functioning
Source: Front Psychol. 2023 Mar 30;14:1120992. doi: 10.3389/fpsyg.2023.1120992 (PMC10097994; doi:10.3389/fpsyg.2023.1120992)
Supplement: Supplementary file 1 [file Data_Sheet_1.docx]

Supplementary Material

The effects of a complex yoga-based intervention on healthy psychological functioning

# Appendix A

**Schedule of the integrative yoga program**

| Week | Activity | Description |
| --- | --- | --- |
| 1 | Yoga session |  |
|  | Club meeting | Sharing experiences of practice, discussion of students’ questions |
| 2 | Yoga session |  |
|  | Scientific lecture | Healthy sleeping habits for hormonal balance |
| 3 | Yoga session |  |
|  | Club meeting | Sharing experiences of practice, discussion of students’ questions |
|  | Scientific lecture | Physiological and psychological effects of nutrition, developing a healthy diet |
| 4 | Yoga session |  |
|  | Scientific lecture | The importance of stress-management and relaxation |
| 5 | Yoga session |  |
|  | Club meeting | Sharing experiences of practice, discussion of students’ questions |
|  | Scientific lecture | The relevance of breathing in stress-management, physiology of breathing |
| 6 | Yoga session |  |
|  | Scientific lecture | The role of asana practice on developing healthy body awareness, effects of asanas on physiology (i.e., cardiovascular, neural, and hormonal functioning) |
| 7 | Yoga session |  |
|  | Club meeting | Sharing experiences of practice, discussion of students’ questions, pranayama practices |
| 8 | Yoga session |  |
| 9 | Yoga session |  |
|  | Club meeting | Sharing experiences of practice, discussion of students’ questions, pranayama practices |
| 10 | Yoga session |  |
| 11 | Yoga session |  |
|  | Club meeting | Sharing experiences of practice, discussion of students’ questions, pranayama practices |
| 12 | Yoga session |  |
|  | Club meeting | Sharing experiences of practice, discussion of students’ questions, pranayama practices |

*Note.* Club meetings did not have a prior topic, they aimed to create time and space for students to share their experiences and address their questions to the teacher. Each session was adapted to the actual needs. After the scientific lectures on breathing (5^th^ week), pranayama practice was also included. Pranayama Series (PS) D and E exercises were taught during these meetings (see below).

**Schedule of the yoga classes**

| Warm up exercises | Neck rotations in sitting pose | 1-2, 3-4, 5-6, 7-8 |
| --- | --- | --- |
|  | Shoulder rotations | 1-2, 3-4, 5-6, 7-8 |
|  | Rabbit pose | 9-10 |
| Supine poses | Leg raises | 1-2 |
|  | Bicycling | 3-4, 5-6 |
|  | Wind relieving pose (with one leg up and with both legs up) | 1-2, 3-4, 5-6, 7-8 |
|  | Dynamic spine twist (PS A) | 1-2, 9-10, 11-12 (6) |
|  | Spine twist | 3-4, 5-6, 7-8 |
|  | Boat pose | 9-10 |
|  | Milling with hand together (fingers interlaced) | 9-10 |
|  | Half bridge (dynamic) (PS A) | 3-4, 5-6, 7-8, 11-12 (7) |
| Sitting poses | One-legged head to knee pose | 9-10 |
|  | Dynamic seated forward bend (Paschimottan asana) | 11-12 (5) |
|  | Butterfly | 9-10 |
|  | Seated spine twist (PS B) | 9-10, 11-12 (8) |
| Kneeling | Dynamic tiger pose (PS B) | 3-4, 5-6, 7-8, 9-10 (as warm up), 11-12 (4) |
|  | Cat-cow (PS B) | 1-2, 3-4 |
| Prone | Cobra pose | 5-6, 7-8, 9-10, 11-12 (9) |
|  | Downward facing dog | 3-4, 5-6, 7-8, 9-10, 11-12 (10) |
| Standing poses | Standing stretch with arms up (PS A) | 1-2 |
|  | Standing side bend with arms up | 1-2, 3-4, 5-6, 11-12 (1) |
|  | Standing forward bend with the help of chair | 1-2 |
|  | Chair pose | 3-4, 5-6, 7-8 |
|  | Warrior 1 | 9-10 |
|  | Dynamic warrior 1 (PS A) | 11-12 (2) |
|  | Chair pose with forward bend (PS A) | 11-12 (3) |
|  | Triangle (Trikonasana) | 7-8, 9-10 |
|  | Warrior 2 | 7-8, 9-10 |
| Inversions | Half shoulder stand | 3-4, 5-6, 7-8, 9-10, 11-12 (11) |
| Belly breathing (PS B) | Lying down with knees up, palms on the belly, 10 breaths | 1-2 |
| Relaxation | Corpse pose (Savasana) 3-5 min | 1-2, 3-4, 5-6, 7-8, 9-10, 11-12 (12) |
| Pranayama Group C | Viloma – interrupted breathing  - Inhale-pause-inhale-exhale-pause-exhale-pause, 5-10 rounds  - Inhale-exhale-pause-exhale; Inhale-pause-inhale-exhale; Inhale-pause-inhale-exhale-pause-exhale, 5-10 rounds each | 7-8  9-10, 11-12 (13) |

*Note*. The intervention consisted of six steps, two classes each. Home practices were based upon these six steps (i.e. six exercise series for home practice). The sequence of the asanas in the two classes of 6^th^ series (i.e. class 11 and 12) slightly differed, thus order numbers are presented in a parenthesis. Pranayama practices were also categorized in six series (PS A-E). PS A (asana holding with breath retention) and PS B (dynamic asanas in which one focused on the place of breathing (hathen)) were practiced during classes with preparations from the first class and with breath retention from class 11 (6^th^ series). PS C involved interrupted breathing (viloma). PS D (breath focus/breath awareness) and PS E (breathing with given timely proportion of inhalation-retention-exhalation-retention (sama vritti, visama vritti) were practiced during Club meetings due to practical considerations.

Besides, students learnt bhramari pranayama and kapalabhati (two well-known pranayama techniques).

# Appendix B

*Descriptive information about the participants*

| Variable | Intervention | | | | Control | | | |
| --- | --- | --- | --- | --- | --- | --- | --- | --- |
|  | Citizen (*n* = 26) | | Teacher (*n* = 18) | | Citizen (*n* = 20) | | Teacher (*n* = 9) | |
| Age (years) (*M, SD*) | 44.80 (8.49) |  | 51.77 (7.68) |  | 45.10 (9.00) |  | 52.56 (8.88) |  |
| Sex (female, *n*) | 20 |  | 18 |  | 15 |  | 9 |  |
| Educational level |  |  |  |  |  |  |  |  |
| higher level (*n*) | 13 |  | 18 |  | 10 |  | 9 |  |
| secondary level (*n*) | 7 |  | 0 |  | 5 |  | 0 |  |
